# Observations on the Present System of Medical Education, with a View to Medical Reform

**Published:** 1834-07-01

**Authors:** 


*Observations on the present System of Medical Education, with a View to Medical Reform.* By A LICENTIATE OF THE ROYAL COLLEGE OF PHYSICIANS OF LONDON.—London, 1834. 8vo. pp. 95.

WERE it our object or duty to amuse our readers, we should make very large extracts from this pamphlet; for it is eminently smart and clever, and not to be laid down again till read through: but, in a Journal like this, consecrated to practical information and stern utility, such a thing is not to be thought of, and we must therefore content ourselves with a couple of short quotations.

" *The Hospital Student.* The short period between the termination of the apprenticeship and the commencement of the hospital career is spent at home, where the young doctor contrives, as much as possible, to convince the natives of the progress he has made in his studies. This is not so difficult a matter with the medical as with most other professions. People can judge of the manual dexterity of a lad, or even of his general mental endowments, but no one can say whether he will be a good or bad doctor.

"The suppositions are, however, in favour of the young student of medicine (for he has dropped the name of apprentice,) from the good testimonials he produces, and his opinion upon a dropsical female, who had been operated upon eight times with success, established his reputation.

" He was decidedly of opinion, that the swelling was caused by a bulk of fluid in the abdomen, and not by pregnancy; and, as she had been tapped already eight times, she might perhaps undergo another operation without endangering her life, though, in reality, life was a thing very ill-defined. It is true, he observed, that this would make the ninth time, and much stress was laid by the ancients upon odd and even numbers, but this opinion stood not the test of modern philosophy. He recommended, in fact, another operation, but would not commit himself by specifying any particular time. It was with great diffidence he gave his opinion, but he thought the constitution might be injured by repeated tappings.

" This opinion gained him great credit, both for his modesty and learning, and his friends thought that the 100*l.* premium had not been thrown away, and his father was the more disposed to part with the money he had put aside for his hospital career, as he saw with pleasure that there was every probability of its being well employed.

" The day arrives for his departure, and, after a little sermonizing about temptations and bad company, and sacrifices, &c. the student finds himself the following day, with his pocket full of money, in Tooley-street, in the Borough. He was now his own master in a private lodging, and every comfort about him; he felt a strange sensation as he counted over the half-notes, for the precaution of cutting them in halves had not been omitted, and he rejoined them with a wafer. He walked up and down his room, which allowed of about three strides in each direction; but it was a palace to him, it was his own, and he could ring his bell as often as he pleased. It was the 29th of September, and the lectures were to commence on the 1st of October; the following day therefore he proceeded to the hospitals, which were situated about five minutes' walk from his lodgings. He meets upon the road a young man with a catheter in his hand, turning into the gates of Guy's, and addressing him, begs he will conduct him to the office for entering to the different courses of lectures. It happened to be a dresser, a kind of non-commissioned officer, and he proceeds with him direct to the apothecary's shop. Here he saw for the first time a hospital laboratory, and he shrugged up his shoulders, when he compared it with his old master's shop. It is in reality an aristocratic establishment; and he who gets one foot into Guy's may hope in time to get both into a carriage, and himself into an apoplexy.

"He was a little astonished at his morning's disbursement, and the more so when he found that he had taken out no tickets for surgery and anatomy, which he came to London to study. He was soon, however, directed to another office, where, upon paying another considerable sum, he became perpetual proprietor of as many courses as he pleased. He had now disbursed almost the whole of the sum with which he was supplied on leaving the paternal house. He returned to his lodgings with the best resolutions, and immediately informed his friends of the legal claims already made upon his purse. The sundries did not hitherto figure in his cash-book; all was fair and legal.

"He had received tickets of admission to each course of lectures. These were printed in ink of various colours, with appropriate Latin mottoes; but he had so far forgotten his Latin as not to be able to construe them, and he determined upon buying a dictionary and grammar, and sticking this winter close to his classics.

"He already regretted his former life, and the frivolous, unprofitable manner in which he passed five years of his existence. He finds himself deficient in the very elements of education, and ignorant of such things as are necessary to his comprehending any part of his medical studies. He is now obliged to go to school again, and devote a portion of that time to elementary education, which is of itself much too limited to teach him what he came to learn." (P. 51.)

"Will patients regret the diminution of draughts and pills, powders and electuaries, the only means at present of giving a medical man food?

"Dr. Paris tells us of an apothecary who declared he put as many ingredients into his mixtures as he well could; for, in shooting out many arrows, it is probable that some will hit.

"Another gave three different kinds of draughts, one to produce heat, the other to produce cold, and the third to modify the effects of the other two. This is the nullifying system with a vengeance; but still the patient had to pay at least 4*s.* 6*d.* for what the prescriber himself confesses could be of no service.

"I had myself an opportunity of witnessing the absurdity of the present system in nearly the first case which I attended. It was a case of confirmed enteritis: neither bleeding nor leeches had been applied, and I attended the case in consultation with the late Dr. T. The apothecary, or rather druggist, who preceded us, had sent draughts, pills, and powders, with the following directions:

"One draught to be taken every two hours.

"One powder every two hours.

"One pill every two hours.

"The stomach rejected every thing taken into it. The patient recovered by bleeding copiously from the arm, and leeches.

"It was a case where no medicine could be taken by the mouth but with disadvantage during a certain stage of the disease; it was, consequently, a case either to starve the apothecary, or destroy the patient.

"Does not such a system need reform? Does not society demand it? Does not the apothecary, in his own defence, call for it?

"The fault is not always with the apothecary; the system leads to great abuses, and sometimes to unavoidable ones. Men of the highest education are found among this body, as well as men of the lowest: let them no longer be confounded together because they both live by charging time and talent upon drugs, and cease to be looked upon as mere tradesmen.

"It is impossible to say how far such innovations may be adopted, or how they will work; but it will be our regret and mortification if they do not tend to the good of all classes of society, should they be put into execution; for I trust the time is near at hand when the satire of the old practitioner will no longer be applicable—

"Juvenis, tua doctrina non promittit opes;
Plebs amat remedia." (P. 93.)

The best part of the work (from which our first extract is taken,) consists of the history of a country apothecary's apprentice: it is, in fact, a medical novel, and ends, as novels ought to do, with the hero's wedding. The apprentice marries his master's daughter, and succeeds to a practice of 150*l.* per annum.

The defect of this book is, that it is rather too smart: there is a perpetual string of antitheses in words and thoughts from beginning to end. We confess, however, that this is not the most unpardonable of faults in our eyes: epigrammatic exuberance resembles its parent, youth, of which the experienced are wont to say, that it is a fault which mends with time; and it must be allowed, on all hands, that wit is a tincture which it is far easier to dilute than to concentrate.

